# The accumulation of active ingredients of *Polygonatum cyrtonema* Hua is associated with soil characteristics and bacterial community

**DOI:** 10.3389/fmicb.2024.1347204

**Published:** 2024-03-15

**Authors:** Qingyou Zhang, Yunzhang Cai, Luping Zhang, Mei Lu, Luyun Yang, Dekai Wang, Qiaojun Jia

**Affiliations:** ^1^College of Life Sciences and Medicine, Zhejiang Sci-Tech University, Hangzhou, Zhejiang, China; ^2^Key Laboratory of Plant Secondary Metabolism and Regulation of Zhejiang Province, Hangzhou, Zhejiang, China

**Keywords:** active ingredient, soil physicochemical properties, soil enzyme activities, bacterial community, correlation analysis, *Polygonatum cyrtonema* Hua

## Abstract

**Introduction:**

With the increasing demand for health products derived from Polygonati rhizoma (PR), people begin to artificially plant *Polygonatum cyrtonema* Hua (*P. cyrtonema*) in the middle and lower reaches of the Yangtze River. To promote *P. cyrtonema* cultivation and increase farmers’ income, efforts are needed to understand the ways to obtain high-quality PR under artificial cultivation conditions.

**Methods:**

Rhizomes of artificial planting *P. cyrtonema* and rhizosphere soils were collected across five regions in Zhejiang Province, China. Subsequently, the contents of the main active ingredients of *P. cyrtonema* and soil properties were analyzed, and both rhizosphere and endophytic bacteria of *P. cyrtonema* were detected by 16S rDNA sequencing. The relationship between the active ingredients and soil properties, and the dominant bacteria were investigated by correlation analysis.

**Results:**

The content of active ingredients of *P. cyrtonema* from the five regions varied significantly, especially polysaccharides and saponins. High-throughput sequencing demonstrated that Proteobacteria was the dominant bacterial phylum in all samples, and *Burkholderia-Caballeronia-Paraburkholderia* was the main endophytic bacterial genus in rhizome. In addition, the bacterial diversity and richness of rhizosphere soil samples were higher than those of rhizome samples. Soil physicochemical properties and enzyme activities were significantly different across regions, leading to notable variations in the community structures of rhizosphere and endophytic bacteria. Redundancy analysis (RDA) displayed that pH and urease (UE) were the major factors altering shifting rhizosphere bacteria community structure. Moreover, the composition and diversity of rhizome endophytic bacteria were principally affected by both soil physicochemical properties and soil enzyme activities. Soil properties and bacteria from rhizosphere soil and rhizome had a considerable impact on certain active ingredients in *P. cyrtonema* under artificial cultivation conditions after Pearson correlation analysis. Polysaccharides were significantly correlated with nutrient-rich soil and endophytic bacteria, such as *Burkholderia-Caballeronia-Paraburkholderia*, *Pseudomonas*, *Ralstonia*, and *Bacillus*. However, flavonoids were associated with nutrient-poor soil. Saponins were positively correlated with OM and available phosphorous (AP) and were significantly negatively affected by rhizosphere bacterial communities.

**Conclusion:**

The study demonstrated that bacterial microorganisms were involved in the accumulation of active ingredients of *P. cyrtonema* together with soil physicochemical properties and enzyme activities, which provided a theoretical basis for the scientific and effective artificial cultivation of high-quality *P. cyrtonema*.

## 1 Introduction

Polygonati rhizoma (PR), a traditional homology of medicine and food in China, is the rhizome of several perennial *Polygonatum* species in the family of *Liliaceae*. There are more than 60 species globally, mainly distributed among the north temperate zone and the north subtropical zone (Tian and Zhao, 2007). Only *Polygonatum sibiricum* Red., *Polygonatum cyrtonema* Hua, and *Polygonatum kingianum* Coll. et Hemsl., were introduced in Chinese State Pharmacopeia (2020). Among these species, *P. cyrtonema* is mainly distributed in the middle and lower reaches of the Yangtze River, including Zhejiang, Anhui, and Jiangxi Provinces. The main chemical components of *P. cyrtonema* include polysaccharides, flavonoids, steroidal saponins, lignans, alkaloids, and anthraquinones (Zhang et al., 2019), among which the first three are the main active ingredients (Chen et al., 2019). Modern pharmacological research demonstrated *P. cyrtonema* had a variety of physiological functions, including anti-tumor, anti-bacterial, hypoglycemic, hypolipidemic, anti-aging, antioxidant, immunomodulatory, and other physiological activities (Yu et al., 2008; Liu J. Y. et al., 2019; Chen et al., 2021; Wang F. F. et al., 2022; Wang Q. L. et al., 2022; Zhang et al., 2023). With a further understanding of the pharmacological effects of *P. cyrtonema* and the improvement of healthcare awareness, the demand for health products derived from *P. cyrtonema* is increasing. Recently, the market gap of *P. cyrtonema* has become increasingly prominent, and the price of *P. cyrtonema* medicinal materials has risen from 2.51 dollars·kg^−1^ (2010) to 8.37–9.77 dollars·kg^−1^, resulting in a rapid decrease in wild *P. cyrtonema* resources. As a result, people begin to artificially plant *P. cyrtonema* to meet market demand. Discovering ways to obtain high-quality *P. cyrtonema* under artificial cultivation conditions will promote the sustainable development of the *P. cyrtonema* essence industry.

Soil is a site where plant rhizosphere bacteria can settle and exchange material energy. Plants interact with the soil and its microorganisms through rhizosphere at all stages of growth, thereby altering the soil’s physicochemical composition and enzyme activity (Wu and Liu, 2022). Soil fertility and plant health are greatly influenced by the microbial population, and soil is thought to play a crucial role in the composition of microorganisms (Diao et al., 2022). Rhizosphere soil microorganisms can improve the ability of Chinese medicinal plants to adapt to the environment, and also increase the content of their active ingredients, thereby affecting the formation of medicinal plants (Guo et al., 2017). Li Q. L. et al. (2023) found that rhizosphere microbiota structure changed dynamically at different growth stages of *Epimedium sagittatum*, and rhizosphere microbes, along with soil physicochemical properties and enzyme activities, participated in the synthesis and accumulation of effective ingredients. The fertilization of fields with decomposed hot pepper stalks improved the quality of *P. kingianum*, changed the structure of rhizosphere bacterial community, and enriched beneficial microorganisms (Wang et al., 2023). However, there are few related studies on the relationship between *P. cyrtonema* and rhizosphere microorganisms.

Endophyte is a microorganism that inhabits plant tissues and takes host plant metabolites as nutrients (Chen et al., 2023). Endophytic bacteria also play a crucial role in plant growth and secondary metabolism (Cui et al., 2022). Wang et al. (2002) demonstrated that the main endophytic Colletotrichum gloeosporioides of *Artemisia annua* increased the amount of artemisinin in *Artemisia annua* L. hairy root culture. Li et al. (2019) isolated two strains of endophytic fungi promoted the accumulation of saponin content in the tissue culture of *P. polyphylla* var. *Yunnanensis*. It was reported that the dominant endophytic fungi like *Setophoma* and *Arbuscular* mycorrhizal in *P. sibiricum* rhizome might be important microbial communities affecting the biosynthesis of the terpene and alkaloid (Fang et al., 2021). Cai et al. (2021) found that the relationship between endophytic microorganisms and active ingredients content was complex, and genera of endophytic bacteria related with polysaccharides, saponins, flavonoids, 5-Hydroxymethylfurfural were identified in *P. cyrtonema* through correlation analysis. Some characteristic endophytic bacteria derived from different origins were significantly correlated with active ingredients of *Eucommiae cortex* (Liang et al., 2023).

Microorganisms and environmental factors are significant contributors to the development of the quality of Chinese herbs as well as their growth and active ingredient accumulation. However, such factors related with the quality of *P. cyrtonema* under artificial cultivation conditions are not yet clear. Therefore, this study analyzed the characteristics of both rhizosphere and endophytic bacteria of *P. cyrtonema* from five regions of Zhejiang Province by 16S rDNA sequencing technology and performed correlation analysis with their active ingredients to identify microorganisms and environmental factors associated with the quality of *P. cyrtonema* under artificial cultivation conditions. The results will provide a theoretical basis for the scientific and effective artificial cultivation of high-quality *P. cyrtonema*.

## 2 Results

### 2.1 Soil physicochemical properties and enzyme activity

The physicochemical properties and enzyme activities of rhizosphere soil in the five plots were significant differences (Table 1). The range of rhizosphere soil pH of *P. cyrtonema* was 4.52–6.81 under different regions, the highest pH (6.81 ± 0.07) recorded on rhizosphere soil was in Qiantang. Organic matter (30.91 ± 2.24 g/kg) was highest in Wuyi. Alkali-hydrolyzable nitrogen (170.94 ± 1.75 mg/kg), available phosphorous (21.95 ± 0.51 mg/kg) and available potassium (272.71 ± 3.38 mg/kg) were significantly high in Jingning. Acid phosphatase (1812.05 ± 36.15 nmol/h/g) and sucrase (13.4 ± 0.11 mg/d/g) in Wuyi were significantly higher than the other four regions. Urease (1368.09 ± 31.26 μg/d/g) was significantly high in Qiantang.

**Table 1:** Statistical table of physicochemical properties and enzyme activities in rhizosphere soil.

Name	Yuhang	Qiantang	Tiantai	Wuyi	Jingning
pH	4.86 ± 0.04c	6.81 ± 0.07a	4.93 ± 0.14c	4.52 ± 0.03d	5.09 ± 0.06b
OM (g/kg)	20.29 ± 1.04c	28.45 ± 0.85ab	27.48 ± 1.40b	30.91 ± 2.24a	28.00 ± 0.90b
AN (mg/kg)	110.27 ± 1.17c	100.10 ± 5.58d	146.95 ± 3.42b	96.83 ± 3.38d	170.94 ± 1.75a
AP (mg/kg)	7.66 ± 0.04b	5.21 ± 0.41d	5.89 ± 0.15c	4.62 ± 0.23d	21.95 ± 0.51a
AK (mg/kg)	175.03 ± 2.39b	67.79 ± 1.15e	162.61 ± 2.85c	88.96 ± 4.48d	272.71 ± 3.38a
ACP (nmol/h/g)	1410.17 ± 16.92b	1320.32 ± 65.20c	1075.13 ± 23.79e	1812.05 ± 36.15a	1224.14 ± 17.42d
UE (μg/d/g)	365.42 ± 20.95d	1368.09 ± 31.26a	402.58 ± 9.46 cd	430.8 ± 19.18bc	467.27 ± 20.78b
SC (mg/d/g)	8.37 ± 0.40c	12.57 ± 0.31b	5.47 ± 0.21d	13.40 ± 0.11a	5.73 ± 0.20d

pH, hydrogen ion concentration; OM, organic matter; AN, alkali-hydrolyzable nitrogen; AP, available phosphorous; AK, available potassium; ACP, acid phosphatase; UE, urease; SC, sucrase. Means with different lower case letters within a row are significant differences (*P* < 0.05).

### 2.2 Quantitative analysis of active ingredients in *Polygonatum cyrtonema*

**Figure 1:**
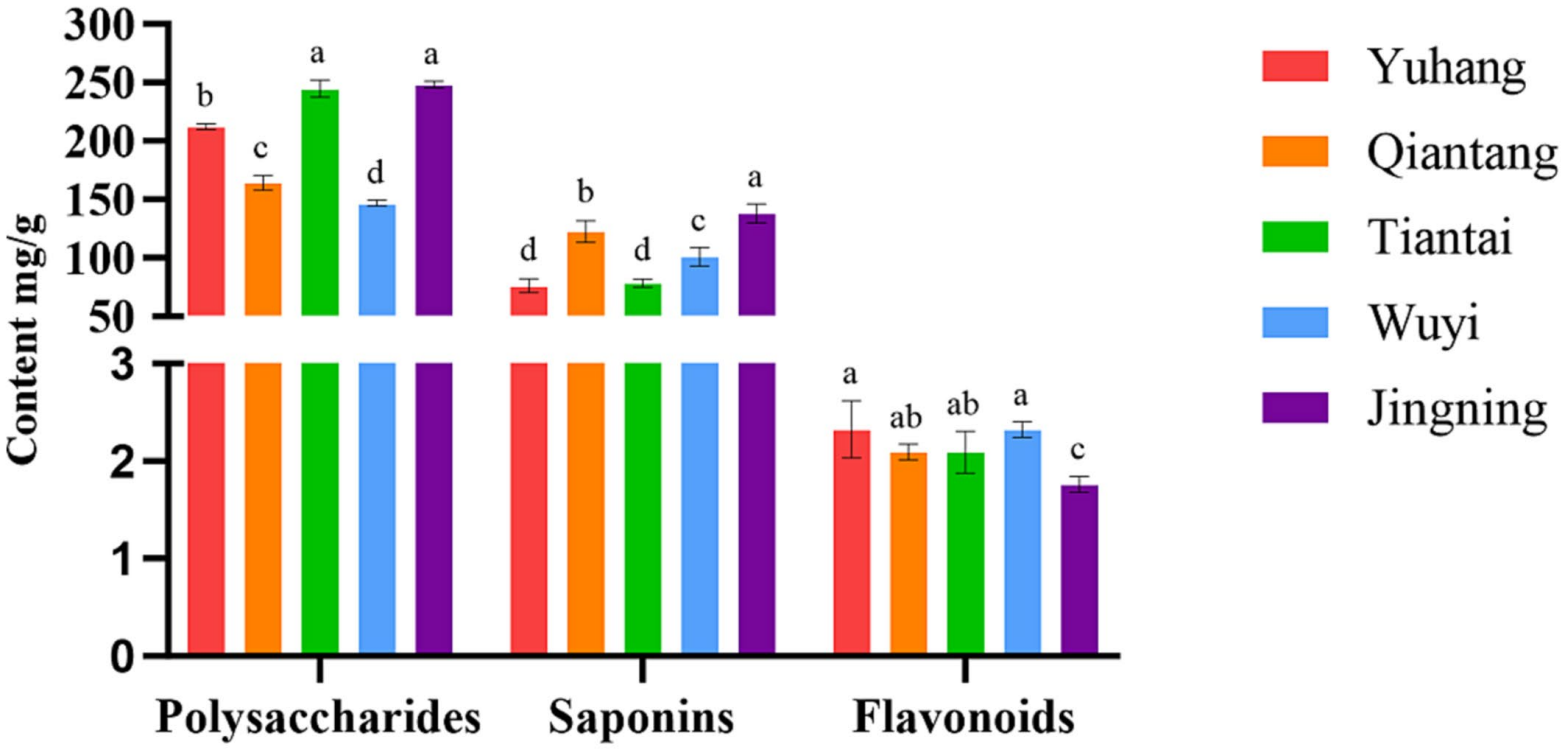
Active ingredient content of Polygonatum cyrtonema in different regions. Means with different lower case letters within a row are significant differences (*P* < 0.05).

The main active ingredients in rhizomes of *P. cyrtonema* from the five plots were detected, and the results were presented in Figure 1. The content of polysaccharides and saponins varied greatly in different regions. The highest concentration of polysaccharides was observed in the plots Tiantai and Jingning, and the lowest polysaccharides were found in Wuyi. The content of saponins was relatively high in the plot Jingning, followed by the plot of Qiantang, Wuyi, Tiantai, and Yuhang. The concentration of flavonoids in Jingning was the lowest, and there was no significant difference in the other four plots. These indicated different patterns of variation in the content of active ingredients of *P. crytonema* under different artificial cultivation conditions.

### 2.3 Analysis of 16S rDNA amplicon sequencing data

#### 2.3.1 Analysis of the OTUs, alpha-diversity, and beta-diversity of *Polygonatum cyrtonema*

**Figure 2:**
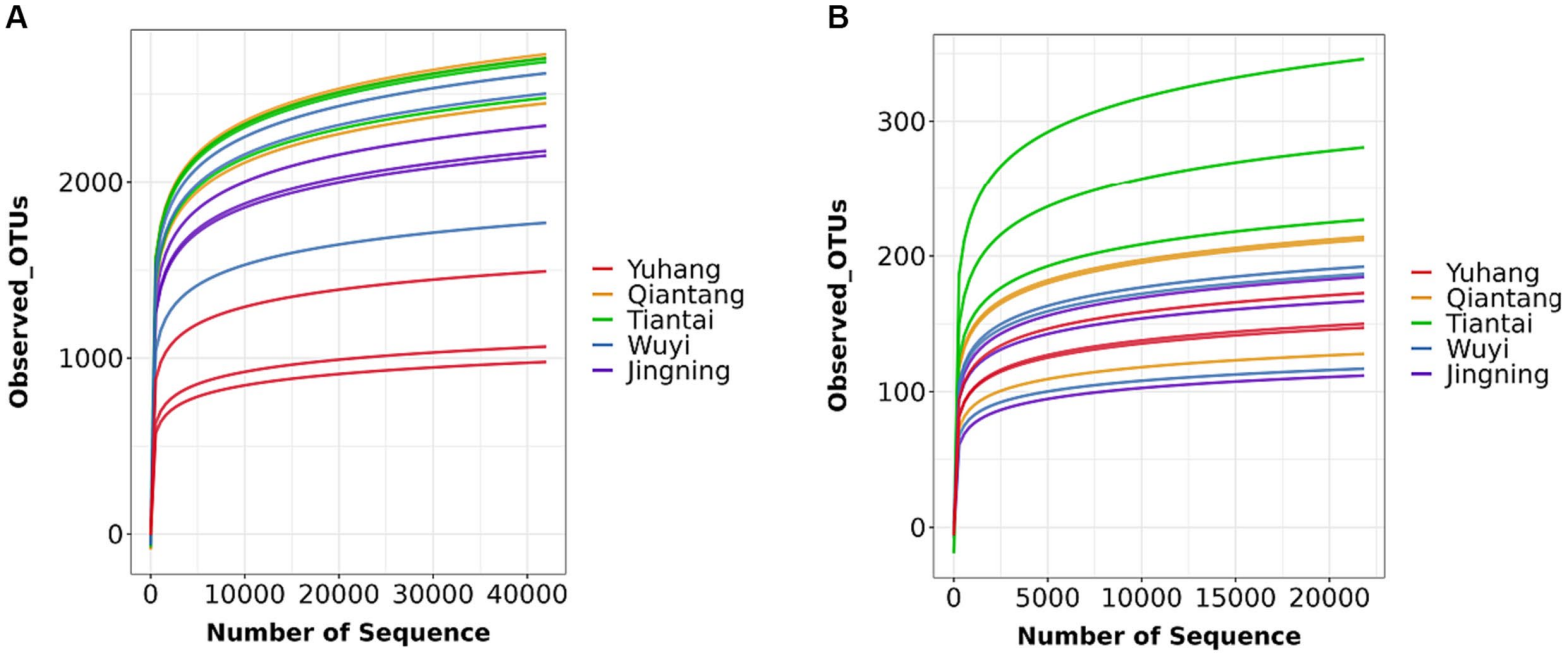
Rhizosphere soil bacteria **(A)** and endophytic bacteria **(B)** rarefaction curves.

**Figure 3:**
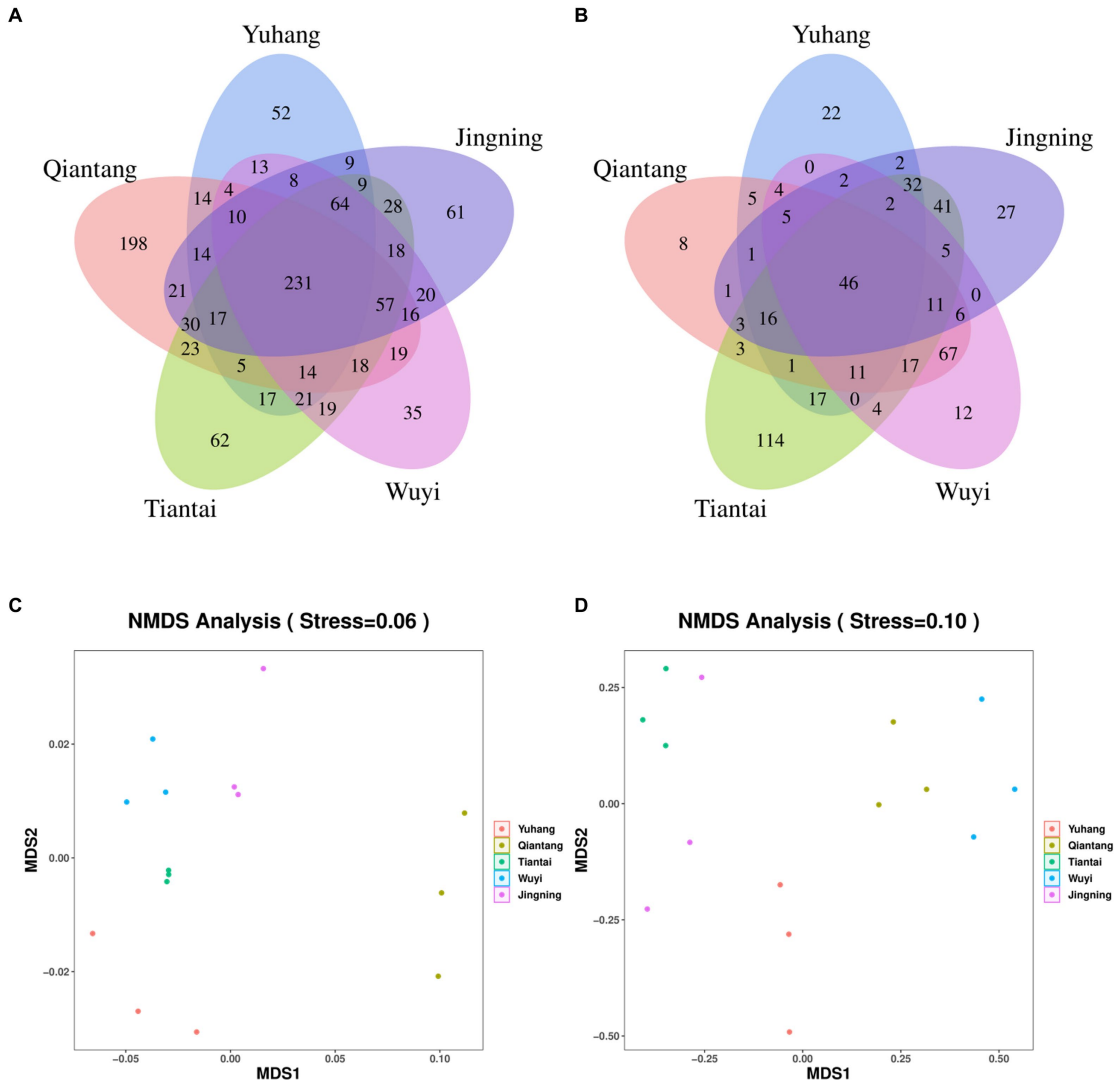
Venn diagrams of rhizosphere soil bacteria **(A)** and endophytic bacteria **(B)** in different regions; Analysis of Beta-bacterial diversity in rhizosphere soil **(C)** and rhizome **(D)** of *Polygonatum cyrtonema* in different origins by NMDS method.

Each sample was analyzed by 16S rDNA amplicon sequencing and the data were summarized. The sequences were clustered into OUTs (100% similarity), and the good coverage of rhizosphere soil or endophytic bacteria included more than 99% (Table 2), respectively. Additionally, along with an increase in the amount of sequencing, the rarefaction curves of all the samples tended to be smooth (Figure 2). Such results indicated that the data amount of sequencing was gradually reasonable and comprehensively reflected the microbial community composition. The Venn diagram was constructed to assess the number of unique and common OTUs across all samples. The number of unique rhizosphere soil bacterial OTUs was 52, 198, 62, 35, and 61 in Yuhang, Qiantang, Tiantai, Wuyi, and Jingning, respectively (Figure 3A). Among them, the number of unique OTUs in Qiantang was the highest, indicating that there were more endemic microbial species. The number of unique endophytic bacterial OTUs accounted for 22, 8, 114, 12, and 27, in Yuhang, Qiantang, Tiantai, Wuyi, and Jingning, respectively (Figure 3B). Tiantai had the highest number of OUTs, and Qiantang had the fewest. In addition, the proportion of common rhizosphere soil bacterial OTUs and endophytic bacterial OTUs was 20.50% and 9.50%, respectively, which indicated that the composition of bacteria in different regions was significantly different.

**Table 2:** Alpha diversity of rhizosphere soil microorganisms and rhizome microorganisms.

Sample name		EffectiveTags	OTU	Shannon	Simpson	Chao1	Coverage (%)
Rhizosphere soil bacteria	Yuhang	70,529 ± 2,012a	1,150 ± 286b	9.25 ± 0.51b	0.997 ± 0.00b	1164.63 ± 269.63b	99.87
	Qiantang	62,587 ± 550c	2,624 ± 172a	10.59 ± 0.01a	0.999 ± 0.00a	2683.42 ± 193.61a	99.63
	Tiantai	70,842 ± 2,208b	2,620 ± 124a	10.62 ± 0.07a	0.999 ± 0.00a	2697.15 ± 151.05a	99.42
	Wuyi	62,965 ± 941c	2,194 ± 92a	10.32 ± 0.14a	0.999 ± 0.00a	2232.61 ± 93.40a	99.67
	Jingning	72,109 ± 2,364ab	2,282 ± 484a	10.39 ± 0.30a	0.999 ± 0.00a	2322.81 ± 501.63a	99.72
Rhizome endophytic bacteria	Yuhang	77,051 ± 1,598a	177 ± 19b	3.38 ± 0.22c	0.811 ± 0.14b	184.03 ± 22.26b	99.95
	Qiantang	67,288 ± 9,621a	196 ± 56b	4.95 ± 0.20a	0.960 ± 0.03ab	196.77 ± 56.90b	99.98
	Tiantai	67,843 ± 11,937a	315 ± 76a	4.50 ± 0.70ab	0.860 ± 0.00ab	250.29 ± 75.81a	99.95
	Wuyi	70,527 ± 15,913a	138 ± 30b	3.54 ± 0.93bc	0.770 ± 0.13b	143.08 ± 26.40b	99.97
	Jingning	72,509 ± 6,631a	173 ± 48b	4.91 ± 0.19a	0.960 ± 0.3a	174.54 ± 49.84b	99.98

Means with different lower case letters within a row are significant differences (*P* < 0.05).

There were differences between rhizosphere and endophytic bacteria diversity. Alpha diversity analysis of microorganisms in rhizosphere soil and rhizome of *P. cyrtonema* was shown in Table 2. The mean OTU abundance, Chao1index, Shannon index, and Simpson index showed that the diversity of the bacterial community in rhizosphere soil was lowest in Yuhang. At the same time, other regions showed no significant difference in the alpha diversity of rhizosphere soil. In addition, the alpha diversity significantly varied along with the sampling plots of rhizomes. The Chao1 index of endophytic bacteria was the highest in Tiantai, while Shannon and Simpson indexes were higher in Qiantang and Jingning. The alpha diversity also demonstrated that the diversity of the bacterial community in rhizosphere soil was higher than that in rhizome. The spatial location map of the samples was obtained by the NMDS method to analyze their β diversity. The results showed that there was little difference between the within-group samples in both rhizosphere soil bacterial and endophytic bacterial community composition (Figures 3C,D).

#### 2.3.2 Comparison of species composition of *Polygonatum cyrtonema* bacterial communities in different regions

**Figure 4:**
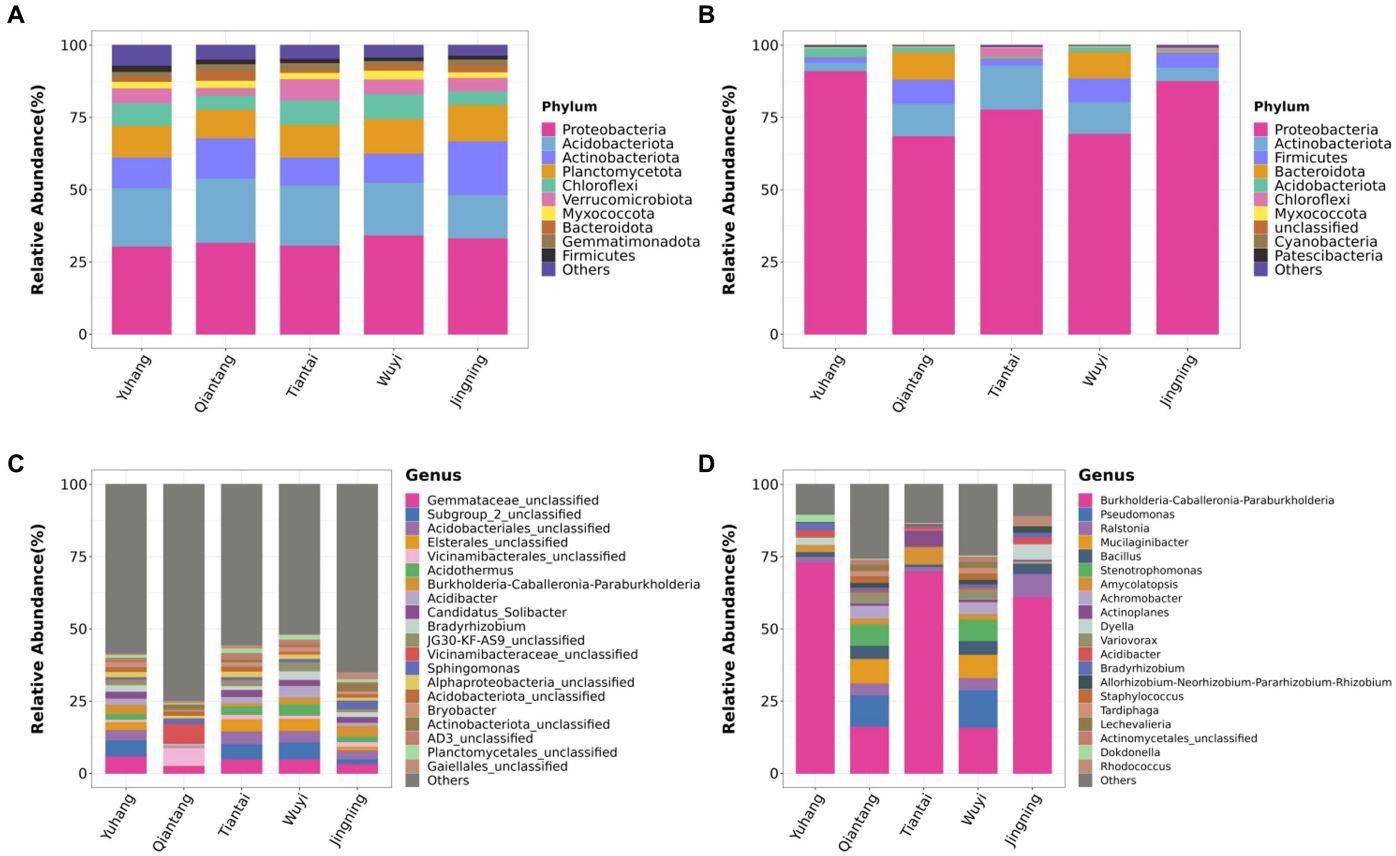
Phylum composition of rhizosphere soil bacteria **(A)** and endophytic bacteria in rhizome **(B)**; Genus composition of rhizosphere soil bacteria **(C)** and endophytic bacteria in rhizome **(D)**.

The rhizosphere soil bacterial composition in different plots was similar at the phylum level. Proteobacteria, Acidobacteriota, Actinobacteriota, Planctomycetota, and Chloroflexi were the most abundant phylum of the *P. cyrtonema* rhizosphere soil (Figure 4A). For the endophytic bacteria, Proteobacteria was the major component of each bacterial community (Figure 4B). The endophytic bacterial composition of rhizomes in Qiantang and Wuyi was similar at the phylum level, while the composition in the other three plots was different (Figure 4B). The top 20 most abundant bacterial genera of rhizosphere soil and endophytic bacteria were selected for further analysis. The rhizosphere soil bacterial composition of Yuhang, Tiantai, and Wuyi was similar but was different in the dominant genus (Figure 4C). The main dominant bacteria of Yuhang was *Gemmataceae*_unclassified, while *Subgroup*_2_unclassified were the abundant bacteria of Tiantai and Wuyi. In addition, *Vicinamibacteraceae*_unclassified and *Burkholderia*-*Caballeronia*-*Paraburkholderia* were the dominant bacteria in rhizosphere soil of Qiantang and Jingning, respectively (Figure 4C). The endophytic bacterial composition of Qiantang and Wuyi was highly similar, whereas their composition of other plots differed (Figure 4D). Additionally, the genus with the highest abundance in rhizome was *Burkholderia*-*Caballeronia*-*Paraburkholderia*, accounting for 73.03%, 16.12%, 69.94%, 15.94%, and 61.13% of the total sequences in Yuhang, Qiantang, Tiantai, Wuyi, and Jingning, respectively.

**Figure 5:**
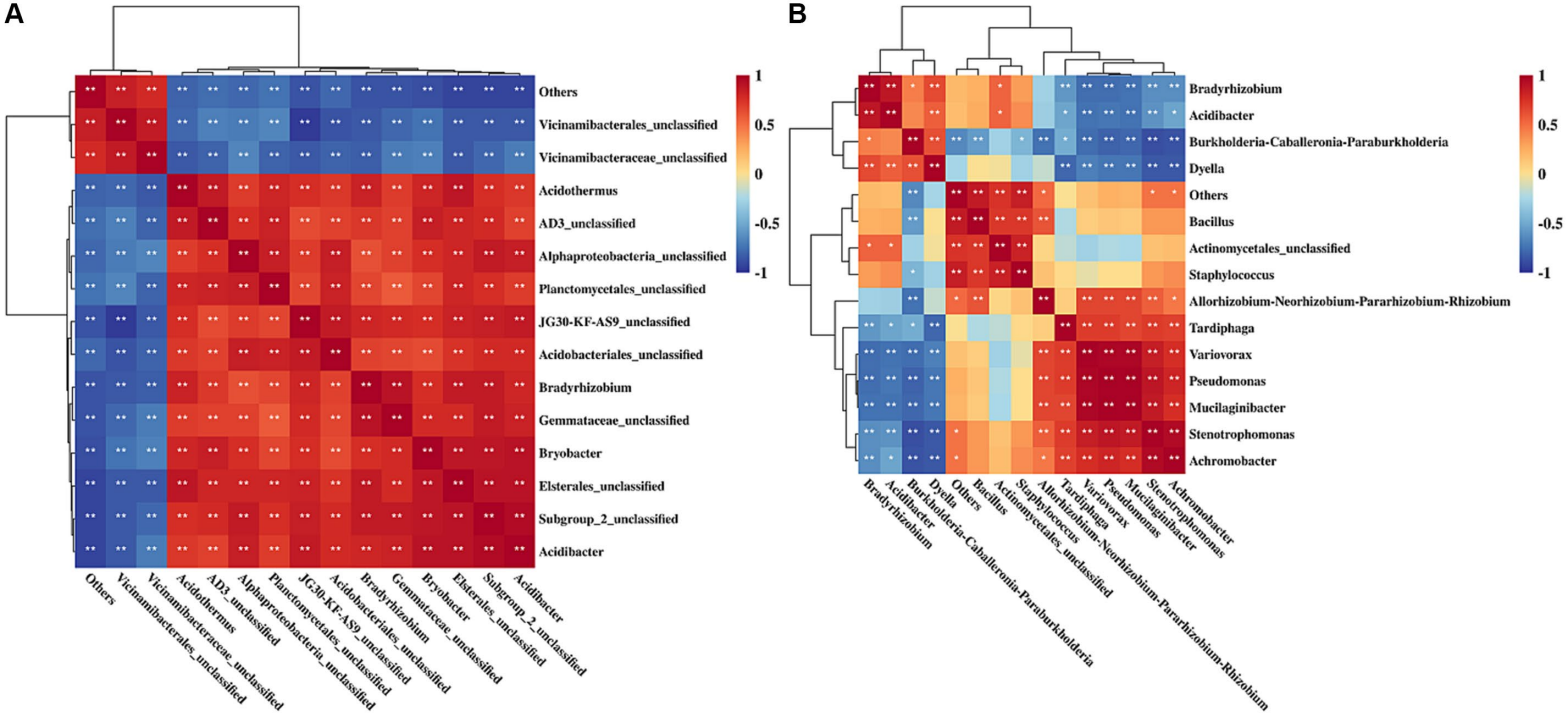
Correlation analysis of rhizosphere soil bacteria **(A)** and endophytic bacteria **(B)** of *Polygonatum cyrtonema*. ^**^p < 0.01; ^*^p < 0.05.

Spearman heatmap analysis of rhizosphere soil differential bacteria showed that 69 pairs of bacteria exhibited significant positive correlations and 36 pairs of bacteria displayed significant negative correlations (Figure 5A). There was a significant positive correlation among 30 pairs of endophytic bacteria and a significant negative correlation among 24 pairs of endophytic bacteria in rhizome (Figure 5B).

### 2.4 Relationship between soil physicochemical properties, soil enzyme activity, bacterial community

**Figure 6:**
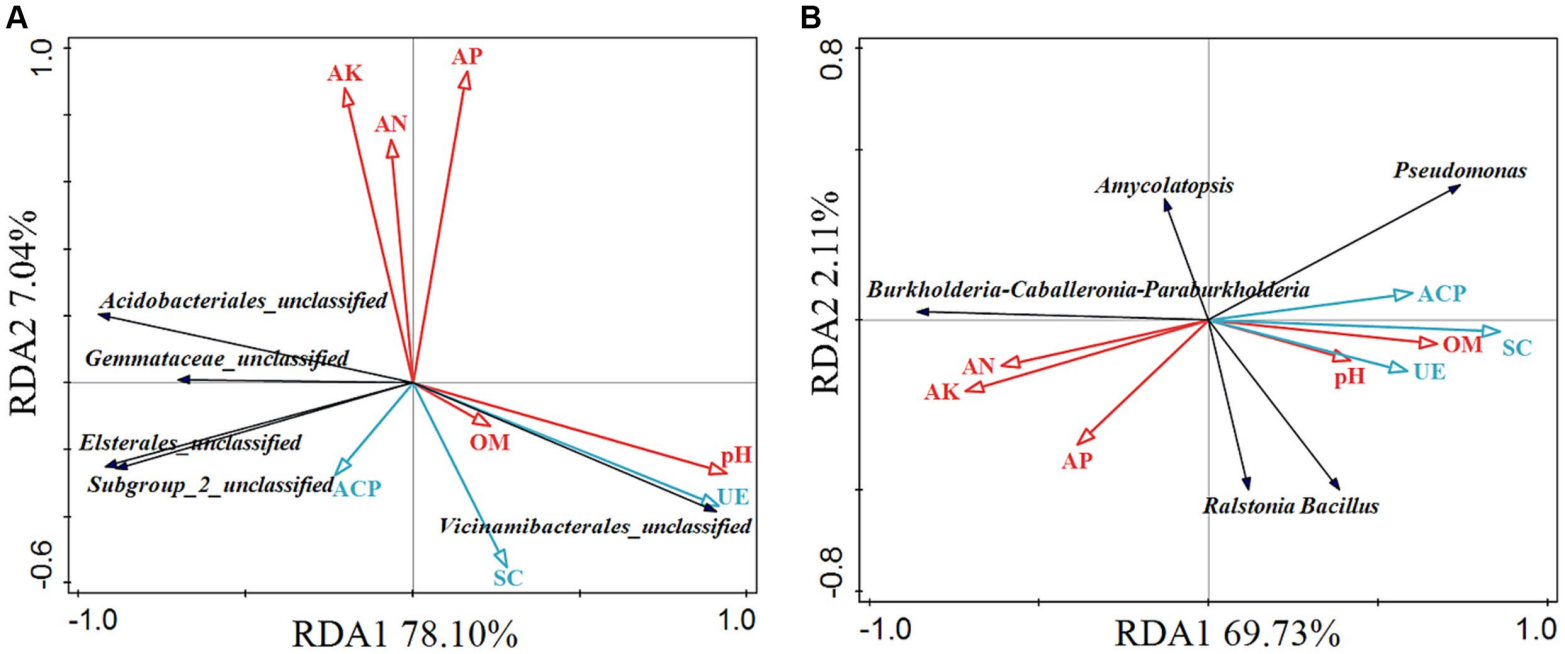
Redundancy analysis (RDA) of rhizosphere soil bacteria **(A)** and endophytic bacteria in rhizome **(B)**. The red arrow and the blue arrow represent the relative position of physicochemical properties and enzyme activity on the horizontal plane. The black arrow represents the species distribution at the genus level, and the longer the arrow, the greater the impact of the species in the sample. Where the angle between the arrow and the sort axis is different, the correlation strength is also different. The smaller the angle, the greater the correlation, and, the longer the length of the arrow, the greater the effect of the environmental factor. pH, hydrogen ion concentration; OM, organic matter; AN, alkali-hydrolyzable nitrogen; AP, available phosphorous; AK, available potassium; ACP, acid phosphatase; UE, urease; SC, sucrase.

Redundancy analysis (RDA) was used to shed light on the influence of variation of soil enzyme activities, and soil physicochemical properties (explanatory variables) on the microbial community (response variables). The rhizosphere soil bacteria RDA analysis showed that the RDA1 was 78.10%, and the RDA2 was 7.04%, which could better reflect the relationship between soil enzyme activities, soil physicochemical properties, and rhizosphere soil bacterial community (Figure 6A). *Gemmataceae*_unclassified and *Acidobacteriales*_unclassified had a certain correlation with AK, AN, and ACP, and were negatively correlated with pH, UE, and OM. *Subgroup*_2_unclassified and *Elsterales*_unclassified had a certain correlation with ACP, SC, and AK, and were negatively correlated with pH, UE, and OM. *Vicinamibacterales*_unclassified was highly correlated with pH and UE and had a certain correlation with SC, and OM. The rhizome endophytic bacteria RDA analysis showed that the RDA1 was 69.73%, and the RDA2 was 2.11% (Figure 6B). *Burkholderia*-*Caballeronia*-*Paraburkholderia* was related to AK, AN, and AP, and negatively correlated with pH, OM, UE, ACP, and SC. *Ralstonia* and *Bacillus* had a certain correlation with pH, OM, AP, UE, ACP, and SC. *Pseudomonas* were related to pH, OM, UE, SC, and ACP, and were negatively correlated with AN, AP, and AK. By comparison, *Amycolatopsis* was not much related to each factor. Pearson correlation analysis revealed that polysaccharides were positively correlated with AN, AP, AK, and *Burkholderia*-*Caballeronia*-*Paraburkholderia*, and were negatively correlated with SC, ACP, *Ralstonia*, *Pseudomonas*, and *Bacillus* (Table 3). The correlation analysis revealed that saponins were negatively correlated with four rhizosphere soil bacterium (*Gemmataceae*_unclassified, *Subgroup*_2_unclassified, *Acidobacteriales*_unclassified, and *Elsterales*_unclassified), and were positively correlated with OM and AP. Besides, flavonoids were negatively correlated with AN, AP, and AK.

**Table 3:** Pearson correlation analysis of active ingredients.

Impact factor		Polysaccharides		Saponins		Flavonoids	
		CC	P	CC	P	CC	P
Soil physicochemical properties	pH	−0.257	0.354	0.442	0.099	−0.237	0.396
	OM	−0.354	0.195	0.520^*^	0.047	−0.160	0.570
	AN	0.890^**^	0.000	0.293	0.289	−0.700^**^	0.004
	AP	0.620^*^	0.014	0.613^*^	0.015	−0.687^**^	0.005
	AK	0.860^**^	0.000	0.205	0.464	−0.537^**^	0.039
Soil enzyme activities	SC	−0.990^**^	0.000	0.112	0.692	0.456	0.088
	UE	−0.465	0.081	0.456	0.088	−0.099	0.724
	ACP	−0.806^**^	0.000	−0.018	0.948	0.509	0.053
Rhizosphere soil bacteria	*Gemmataceae*_unclassified	0.140	0.618	−0.743^**^	0.002	0.505	0.055
	*Subgroup*_2_unclassified	0.034	0.904	−0.751^**^	0.001	0.482	0.069
	*Acidobacteriales*_unclassified	0.428	0.112	−0.562^*^	0.029	0.157	0.575
	*Elsterales*_unclassified	0.052	0.853	−0.652^**^	0.008	0.334	0.224
	*Vicinamibacterales*_unclassified	−0.380	0.163	0.428	0.112	−0.133	0.636
Endophytic bacteria in rhizome	*Burkholderia-Caballeronia-Paraburkholderia*	0.953^**^	0.000	−0.288	0.415	−0.333	0.225
	*Ralstonia*	−0.600^**^	0.018	0.177	0.528	0.051	0.856
	*Pseudomonas*	−0.566^*^	0.031	0.236	0.396	0.078	0.781
	*Bacillus*	−0.853^**^	0.000	0.159	0.572	0.358	0.190
	*Amycolatopsis*	−0.022	0.938	−0.280	0.311	0.246	0.377

*Significant correlation (*P* < 0.05);**Extremely significant correlation (*P* < 0.01).

## 3 Discussion

The quality of medicinal plants was closely related to the ecological factors in growing locations, and the active ingredients and biopotency reflected their quality (Liu Y. J. et al., 2019). Microorganisms directly or indirectly participated in the growth and development, metabolism, and active ingredient accumulation of medicinal plants in a variety of ways (He et al., 2020). The active ingredients of artificially planted *P. cyrtonema* in different regions varied greatly, especially polysaccharides and saponins (Figure 1). It was reported that polysaccharides (7.48%~15.23%) and saponins (1.82%~6.49%) contents of *P. sibiricum* from different origins were significantly different (Qian et al., 2022). He et al. (2022) found that the different growth environments led to differences in polysaccharide contents of *P. sibiricum*, with comparatively higher levels of polysaccharides produced in Yunnan and Hebei Province. Polysaccharides contents of the five regions were more than 14.68% (Figure 1), which was more than twice the amount specified in State Pharmacopeia (2020), indicating that artificial planting was beneficial for the accumulation of such active ingredients. Jiao et al. (2016) also reported that all the new rhizome polysaccharides of *Polygonatum* Mill. were increased and met the Chinese Pharmacopoeia standards limits after the artificial planting in *Polygonatum* Planting Base of Buchang Pharma Group, Lueyang County, Shaanxi Province. Considering the effect of the environment on the active ingredients of *P. cyrtonema*, it was feasible that artificial cultivation might provide suitable growth conditions to accumulate active ingredients, especially polysaccharides.

High-throughput sequencing showed that rhizosphere and endophytic bacteria of different regions were abundant, and the number and species of rhizosphere bacteria were higher than those of endophytic bacteria (Table 2). Wang Y. B. et al. (2022) found that the diversity of bacteria in *Cinnamomum camphora* (L.) Presl rhizosphere soil was significantly higher than that of endophytic bacteria in plant organs such as camphor roots. At the phyla level, rhizosphere bacteria groups of *P. cyrtonema* were dominated by Proteobacteria, Acidobacteria, Actinobacteriota, Planctomycetota, and Chloroflexi (Figure 4A). Both Proteobacteria and Chloroflexi were also the major bacteria identified in rhizosphere soil of *P. kingianum* (Wang et al., 2023). The dominant phylum of endophytic bacteria groups was Proteobacteria (Figure 4B), which was also identified in the endophytic bacteria community of *P. cyrtonema* from Xinhua, Hunan Province (Cai et al., 2020). At the genus level, most of the bacteria in rhizosphere soil were unclassified, indicating that rhizosphere soil samples contained a large number of unknown microbial species (Figure 4C), which was also reported in the study of *Cynanchum bungei* Decne (Li et al., 2022), *Asarum heterotropoides* F. Schmidt var. *mandshuricum* (Maxim.) Kitag (Yu et al., 2022), and *Phyllostachys edulis* (Yuan et al., 2023). These studies showed that the most of rhizosphere bacteria at the genus level were not identified and studies of rhizosphere bacteria might be useful to improve our knowledge of their behavior and effects on the accumulation of active ingredients in medicinal plants. *Burkholderia-Caballeronia*-*Paraburkholderia* was the dominant genus of endophytic bacteria and also demonstrated relatively high abundance in rhizosphere soil (Yuhang, 3.24%; Qiantang, 0.17%; Tiantai, 1.07%; Wuyi, 2.32%; Jingning, 3.73%; Figure 4D). It seemed that endophytic bacteria might transmit via either a horizontal pathway (i.e., obtained from the vicinal environment) or vertically (i.e., gained directly from the parent) (Shade et al., 2017), due to its abundance in rhizosphere soil (Figure 6B). The results indicated that the relationship between rhizosphere soil bacteria genera was mainly mutualistic and symbiotic, while the correlation between endophytic bacteria genera was not as close as that between rhizosphere soil bacteria genera (Figure 5). According to research by Xie et al., most of the rhizosphere bacterial genera of *Angelica sinensis* at various growth stages exhibited a positive association, suggesting that rhizosphere soil bacteria were more likely to coexist in a mutually beneficial symbiotic relationship (Xie et al., 2023).

Soil environmental factors and soil enzyme activity were important indicators of the natural environment, which determined the composition of soil microbial communities (Gong et al., 2020; Ma et al., 2021). RDA analysis showed that both rhizosphere soil bacteria (*Vicinamibacterales*_unclassified) and endophytic bacteria (*Pseudomonas*, *Bacillus*, and *Ralstonia*) were associated with pH (Figure 6). Jiang et al. (2023) found that pH remarkably affected the bacterial community assembly in the tobacco rhizosphere. Soil enzyme activity also correlated with bacterial community structure, the rhizosphere bacteria *Vicinamibacterales*_unclassified had a significant positive correlation with UE, while the other four rhizosphere bacterium were negatively correlated with UE (Figure 6A). According to the research of *Angelica sinensis* by Xie et al. (2023), there was a significant positive correlation between UE and *Bacteroides* and *Chaetomium* in the rhizosphere, and UE was negatively correlated with other bacteria, such as *Marseillia*. The soil enzyme activity was negatively correlated with the endophytic bacteria *Burkholderia*-*Caballeronia*-*Paraburkholderia* and correlated with *Pseudomonas*, *Bacillus*, and *Ralstonia* (Figure 6B). Besides, bacterial communities were influenced by AN, AP, and AK, with endophytic bacteria being more susceptible to soil properties than rhizosphere bacteria (Figure 6), which was consistent with the results by Li J. Q. et al. (2023). However, the AN, AP, and AK were the main driving forces of *P. kingianum* rhizosphere bacterial community structure by chemical fertilizer treatment (Wang et al., 2023), possibly due to the higher AN, AP, and AK content in the cultivation of *P. kingianum* compared to our research. Moreover, Liu et al. (2022) found that rhizosphere bacteria of *Lycium barbarum* fruit positively correlated with soil pH, monthly average atmospheric humidity and monthly average soil humidity. All of these suggested that soil characteristics played important roles in the selection of plant microbiomes (Gupta et al., 2021). Therefore, soil physicochemical properties and enzyme activities of rhizosphere soil microorganisms, which was also demonstrated in *Stellera chamaejasme* L. (Cheng et al., 2022).

For medicinal plants, the ecological environment factors of their origin were closely related to the quality and authenticity of Chinese medicinal plants (Li Q. L. et al., 2023). The Pearson correlation analysis showed that the active ingredients were greatly affected by bacterial communities and soil properties of *P. cyrtonema*. Polysaccharide was strongly correlated with soil physicochemical properties, enzyme activity, and endophytic bacteria (Table 3). The significant positive correlation between polysaccharides and AN and AP was also reported in *Linze Jujube* (Tian et al., 2023). In the present study, *Burkholderia*-*Caballeronia*-*Paraburkholderia* was positively correlated with polysaccharide content, while most of the dominant bacterial groups were negatively correlated with polysaccharide content, which might be related to the metabolic consumption of endophytic bacteria based on polysaccharides and other carbohydrates (Cai et al., 2020, 2021). Saponins were significantly positively correlated with OM and AP, and negatively correlated with four rhizosphere bacterial communities (Table 3). The saponins of ginseng under three matrix combinations were significantly positively correlated with soil AP (Fang et al., 2022). There was a positive correlation between saikosaponin and OM content of *Bupleurum chinense* in different habitats (Liu et al., 2020). Studies showed that the soybean field soil bacterial α-diversity was decreased with four different saponin treatments (Nakayasu et al., 2021). In addition, our results showed that flavonoids were negatively affected by soil physicochemical properties (Table 3). Studies displayed that the content of total *Epimedium koreanum* flavonoids was negatively related to AN (Liu et al., 2021). Li et al. (2017) found that the AK had an inhibitory effect on the flavonoid content of *Caulis Spatholobi*. It was reported that abiotic stress such as temperature, salt, and UV radiation could enhance the total flavone synthesis in *Sarcandra glabra* (Thunb) Nakai (Su and Zhou, 2009). Therefore, nutrient deficiencies might also promote *Polygonatum* flavonoids accumulation. Consequently, *P. cyrtonema* from Jingning showed the highest content of polysaccharides and saponins (Figure 1), because Jingning plot had a higher level of AN, AP, and AK and a higher abundance of *Burkholderia*-*Caballeronia*-*Paraburkholderia* (Table 1; Figure 4D). On the contrary, Wuyi had the lowest polysaccharides concentration and a relatively high flavonoids concentration than other regions, possibly due to its poor quality of the soil nutrients (Table 1; Figure 1). Our study demonstrated that bacterial microorganisms were involved in the accumulation of active ingredients of *P. cyrtonema* together with soil physicochemical properties and enzyme activities, which provided a theoretical basis for the scientific and effective artificial cultivation of high-quality *P. cyrtonema*.

## 4 Materials and methods

### 4.1 Material collection and preparation

**Figure 7:**
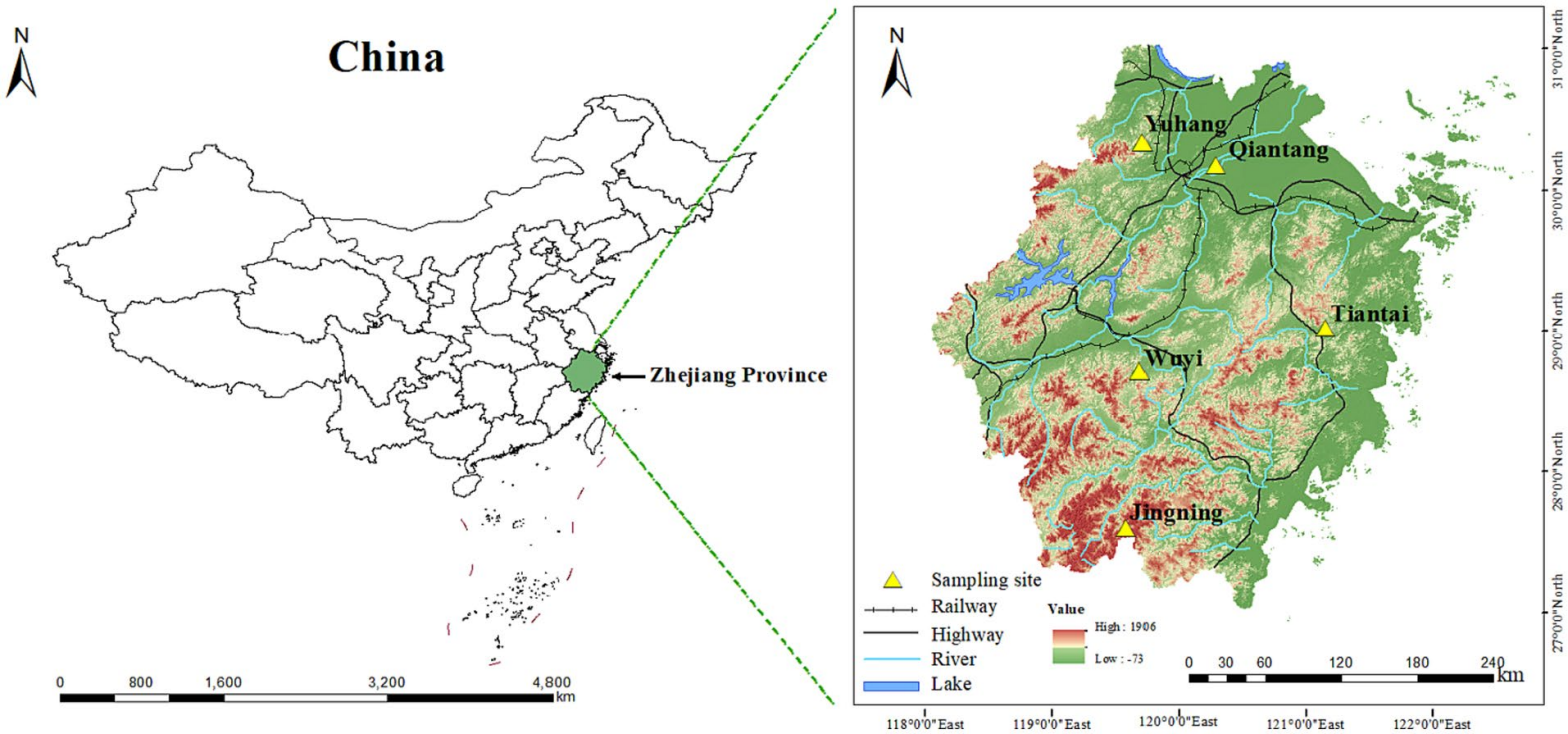
Map of sampling sites in Zhejiang, China. The map was plotted by Arcgis (Version 10.8, Esri, United States).

*Polygonatum cyrtonema* originated from Huangshan City, Anhui Province was planted in Yuhang, Qiantang, Tiantai, Wuyi, and Jingning in Zhejiang Province in November 2019, respectively. All the samples were collected in November 2022 (Figure 7). Five representative plants were collected from each region as sampling plants and were divided into two parts. After being thoroughly cleaned and sanitized, a part of rhizomes was divided into three subsamples and refrigerated at −80°C for high-throughput sequencing of endophytic bacteria. The other parts were used to determine the content of active ingredients after drying at 55°C and passed through a 60 mesh sieve. The rhizosphere soil (at 2 mm to rhizomes, and at 5–10 cm depth from the soil surface) of each region was collected by sterile brush and mixed as a sample. Soil samples were transported to the laboratory in an icebox, and each sample was further divided into two subsamples. One subsample of rhizosphere soil was stored at −80°C for high-throughput sequencing of rhizosphere bacteria, and the other was used for physicochemical properties and enzyme activities analysis after naturally drying and passed through a 2 mm sieve. The sample names of rhizomes and rhizosphere soil corresponded one by one.

### 4.2 Soil physicochemical properties and enzyme activity analysis

Soil physicochemical properties were determined according to the methods in “Laboratory Analysis Methods for Soil Investigation” (Zhang and Gong, 2012), including pH value (laboratory pH meter), organic matter (the external heating method of concentrated sulfuric acid and potassium dichromate), alkali-hydrolyzable nitrogen (alkali hydrolysis diffusion method), available phosphorus (sodium bicarbonate leaching molybdenum-antimony anti-colorimetric method), and available potassium (ammonium acetate leaching flame photometer method). Soil urease, soil saccharase, and soil acid phosphatase were measured by soil enzyme kits (Beijing Solarbio Science & Technology Co., Ltd., Beijing, China), and a Multimode reader (Synergy HTX, BioTek Instruments, Inc., United States).

### 4.3 Quantification of active ingredients in *Polygonatum cyrtonema*

The anthrone-sulfuric acid method was used to determine the polysaccharides content (State Pharmacopeia, 2020). The content of total flavonoids was determined according to Chen et al. (2012). The total saponins were measured as described by You et al. (2010).

### 4.4 Bacterial community profiling by 16S rDNA amplicon sequencing

DNA from rhizosphere soil and rhizome samples was extracted using the DNA Kit (D4015-02, Omega, Inc., United States) according to the manufacturer’s instructions. The reagent designed to uncover DNA from trace amounts of the sample was effective for the preparation of DNA of most bacteria. Nuclear-free water was used for blank. The total DNA was eluted in 50 μL of Elution buffer and stored at −80°C until analysis by LC-Bio Technology Co., Ltd., Hang Zhou, Zhejiang Province, China (Zhang Q. Q. et al., 2018; Zhang X. Y. et al., 2018; Chen et al., 2019). PCR amplification of 16S rDNA high variable V3/V4 region of bacteria was performed using 341F (5′-CCTACGGGNGGCWGCAG-3′) and 805R (5′-GACTACHVGGGTATCTAATCC-3′) primers (Xiong et al., 2012; Sundberg et al., 2013; Xu et al., 2016). All the amplicons were sequenced using Illumina NovaSeq PE250 (PE250, CA, United States) high-throughput sequencing technology, and bioinformatics analysis of sequences was performed using software such as QIIME2 (Bolyen et al., 2019).

### 4.5 Data processing and analysis

After sequencing, paired-end reads were assigned to samples based on their unique barcode and truncated by cutting off the barcode and primer sequence. Paired-end reads were merged using FLASH (Magoc and Salzberg, 2011). Quality filtering on the raw reads was performed under specific filtering conditions to obtain high-quality clean tags according to the fqtrim (Version 0.94, CCB, United States). Chimeric sequences were filtered using Vsearch (Rognes et al., 2016). After dereplication using DADA2 (Callahan et al., 2016), a feature table and feature sequence were obtained. Alpha diversity and beta diversity were calculated by normalizing to the same sequences randomly. Then according to the SILVA (Quast et al., 2013) classifier, feature abundance was normalized using the relative abundance of each sample. Alpha diversity was applied in analyzing the complexity of species diversity for each sample through 5 indices, including Chao1, Observed species, Goods coverage, Shannon, and Simpson, which were calculated with QIIME2. Beta diversity was calculated by QIIME2, and the graphs were drawn by the R package (R Core Team, 2019). Blast (Boratyn et al., 2019) was used for sequence alignment, and the feature sequences were annotated with the SILVA database for each representative sequence. Other diagrams were implemented using the R package.

The data of soil physicochemical properties, soil enzyme activities, and active ingredients were checked by IBM SPSS Statistics 25.0 (SPSS Inc., Chicago, IL, United States) to test whether they met the normal distribution, and then conducted a differential analysis. The content of active ingredients was plotted by GraphPad Prism (Version 9.5, GraphPad, United States). Redundancy analysis (Desarbo et al., 2016) in Canoco5.0 (Version 5.0, Microcomputer Power, United States) was used to explore the correlation among rhizomes endophytic bacteria or rhizosphere soil bacteria, soil physicochemical parameters, and enzyme activities (Cheng et al., 2022). A Pearson correlation analysis (Pearson, 1980s) with the SPSS 25.0 Software was used to study the relationship between active ingredients of *P. cyrtonema* rhizomes and rhizomes endophytic bacterial community index, rhizosphere soil bacterial community index, soil physicochemical parameters, and enzyme activities.

## Data availability statement

The datasets presented in this study can be found in online repositories. The names of the repository/repositories and accession number(s) can be found at: NCBI—PRJNA1071871.

## Author contributions

QZ: Writing – original draft, Conceptualization, Data curation, Formal analysis, Investigation, Methodology, Validation, Visualization. YC: Conceptualization, Investigation, Writing – original draft. LZ: Formal analysis, Writing – original draft. ML: Methodology, Visualization, Writing – original draft. LY: Investigation, Methodology, Writing – original draft. DW: Supervision, Validation, Writing – review & editing. QJ: Project administration, Resources, Supervision, Validation, Writing – review & editing.
